# Supplementary material for: The Impact of Different Types of Rice and Cooking on Postprandial Glycemic Trends in Children with Type 1 Diabetes with or without Celiac Disease
Source: Nutrients. 2023 Mar 29;15(7):1654. doi: 10.3390/nu15071654 (PMC10096979; doi:10.3390/nu15071654)
Supplement: Supplementary file 1 [file nutrients-15-01654-s001.zip › nutrients-2286742-supplementary.pdf]

.

Supplementary Table S1. Glycemic trends after consumption of risotto white rice, boiled white rice, and boiled black rice over the following 12 hours in T1D children. In particular, glucose values were evaluated as the average values of the 12 measurements recorded each hour. Data are expressed as mean ± SD.

	Risotto white rice	***Boiled White rice***	***Boiled black rice***
Glu 0	177,1±79,2	150,1±88,9	120,1±40,7
Glu 1	209,5±68,8	197,1±89,9	135,6±45,8
Glu 2	231,4±35,3	187,9±37,6	164,6±44,5
Glu 3	244,7±59,6	191,9±46,5	143,4±35,7
Glu 4	236,7±70,8	156,2±70,2	129,7±29,8
Glu 5	205,9±61,9	133,9±104,3	126,7±20,5
Glu 6	157,9±60,7	140,6±88,7	139,5±42,1
Glu 7	136,5±38,7	145,2±58,3	140,8±33,9
Glu8	129,9±15,9	139,4±39,7	125,7±27,2
Glu 9	123,1±20,4	139,8±24,7	139,2±29,3
Glu 10	128,5±17,4	139,1±22,3	134,1±21,2
Glu 11	126±18,4	117,9±37,5	146,5±26,9
Glu 12	131,6±21,1	111±26,8	145,5±28,1
p for trend	***<0,001***	***0.02***	***NS***
